# Cancer control policy in Australia

**DOI:** 10.1186/1743-8462-3-12

**Published:** 2006-10-23

**Authors:** Cleola Anderiesz, Mark Elwood, David J Hill

**Affiliations:** 1 The Cancer Council Victoria, Carlton, Victoria, Australia; 2 Cancer Control Research Program, BC Cancer Agency formerly Director, National Cancer Control Initiative, Vancouver, British Columbia, Canada

## Abstract

Australia has an evolving national cancer control agenda. In this paper, we review the history and development of cancer control policies in Australia up to the end of 2005, and discuss the principal publications produced by both government and non-government groups which have given rise to cancer control recommendations, goals and targets. These cancer control plans have arisen in response to the impact of cancer on the Australian community and in recognition of the health gains that can be made through effective cancer control. They have been developed either in the context of a broader framework of health policy or as specific endeavours in regard to cancer. The specific recommendations and strategies proposed have focused on reducing the impact of cancer in the Australian population. Most commonly, recommendations, goals, and targets within the cancer control plans have addressed points along the continuum of care, specific cancers, and frameworks and processes. The strength of these reports is their comprehensive approach in identifying priority cancers and areas where specific developments should impact on morbidity and mortality. In the future, cancer control plans should be better supported by economic evaluations, and greater financial support for implementation and regular assessment is needed to identify progress on cancer outcomes. The more recent national and State cancer control plans include the development of frameworks to foster a coordinated and cohesive approach to the delivery of cancer care. These plans represent important reforms in cancer care in Australia, and have the potential to reduce the impact of cancer on the community and improve health outcomes.

## Background

### The burden of cancer, and cancer control

Cancer has a great impact on the Australian community. One in three men and one in four women will develop cancer by the age of 75. In 2001, there were over 88,000 new cases of cancer (excluding non melanoma skin cancer), and over 36,000 deaths attributable to cancer in Australia [1], in a population of approximately 19.6 million people. In addition, the estimated number of treated cases of non-melanoma skin cancer in Australia in 2002 was 347,000 [2]. The number of new cancer cases is increasing, as is the number of people living with the diagnosis of cancer [3]. Between 1990 and 2000 there was a 36% increase in new cancer cases, but only 12% population growth. The annual age standardised incidence rate (using the WHO world standard population) is 338 per 100,000 people and the risk of developing a cancer before the age of 75 is 1 in 3 [1]. It is estimated that over 247,000 people are living in the Australian community with a cancer diagnosed up to five years earlier (again excluding non-melanoma skin cancer) [4]. The impact of cancer is far reaching. A diagnosis of cancer affects not only the individual but also their family, the community, and the health system. The estimated annual health expenditure due to cancer in 2000–2001 in Australia was $2.7 billion [5], 5.5% of total health care expenditure.

Cancer control is defined as: *"all actions that reduce the burden of cancer in the community. It includes every aspect of care, from prevention and early detection to curative treatment and palliative care, all underpinned by the best scientific evidence available" *[6].

Cancer control activities include public education, advocacy, research, early diagnosis and screening, specific cancer therapy, patient care, and support and palliative care, and so involve hospital and community health care providers and also a strong voluntary sector led by the Cancer Councils.

The development of national cancer control strategies and plans did not commence in earnest until the 1980s. Over the last 18 years several reports have been produced identifying national priorities for cancer control in Australia and strategies to reduce the burden of cancer. We will review the major developments focusing particularly on plans aimed at fostering integrated and coordinated cancer control and improving outcomes. We will concentrate on strategic approaches to cancer control, for all cancers, at the national level. We will not discuss in any detail the many important overlapping strategies that relate to more than cancer, such as the National Tobacco Strategy [7] and the National Palliative Care Strategy [8], or other developments specific to one type of cancer or one component of cancer care. In particular, the development with Federal Government funding of the National Breast Cancer Centre in 1995 has led to rapid developments in the breast cancer area; and there have been many specific reports on the provision of radiotherapy services and on the oncology workforce, which will not be covered here. Also relevant to cancer control is the development, mainly by the Australian Cancer Network (a voluntary association of cancer specialists), of evidence-based clinical practice guidelines for most cancers. Most of these guidelines have gone through the extensive review and approval process of the National Health and Medical Research Council (NHMRC). The existing publicly funded screening programs for breast cancer using mammography (established 1982), and for cervical cancer using Pap smears (established 1991) are described in their own reports [9,10], as is the pilot phase of the development of a screening program for colorectal cancer [11]. Developments at State and Territory level have progressed rapidly, particularly in New South Wales [12] and Victoria [13], and most other States are developing cancer control programs.

### A historical review – cancer control in the broader framework of health policy

Australia has a complex healthcare system. The Australian (Federal) Government subsidises out of hospital medical services through its Medicare system. The Federal Government also has direct responsibility for the breast and cervical screening programs, the Pharmaceutical Benefits Scheme, and some other areas such as the provision of radiotherapy services and aged care. Hospital care and some community services are the responsibility of State and Territory Governments and these are publicly funded with private services supported by health insurance plans. Cancer care and cancer control have a large component of non-government and non-profit (voluntary) support, particularly in regard to education, prevention, and the psychosocial support of cancer patients, their families and carers. The national strategic approaches that will be described in this paper originate from either the government or non-government sectors. Several include input from both government and non-government groups, along with consumers and health professionals. The major non-government voluntary organisations are the Cancer Councils, based in each State and Territory with a national office, The Cancer Council Australia, which was formerly known as the Australian Cancer Society. Consumer input has been of increasing importance as exemplified by the contribution of many different organisations, dealing with all cancers, such as Cancer Voices New South Wales, and also groups dealing with one type of cancer.

#### Health for all Australians, 1987

In 1987 the Australian Health Ministers' Advisory Council (AHMAC) established the Health Targets and Implementation (Health for All) Committee with the aim of developing national health goals and targets to reduce inequalities in health status. It was acknowledged that while by world standards the health of Australians was very good, there was a high prevalence of particular illnesses, and in a number of areas, health outcomes could be improved. At this time in Australia, cancer was the second most common cause of death and the cancer death rates were increasing on an age-adjusted basis. The top five cancers ranked on the basis of the number of new cases and the years of life lost were: lung cancer, breast cancer, colon cancer, prostate cancer, and melanoma.

It was recognised that these cancers could be influenced by primary or secondary prevention strategies. The *Health For All Australians *report [14] recommends nine goals and 15 targets relating to these cancers and to tobacco smoking. These cancer goals, targets and strategies were initially proposed by the Australian Cancer Society as part of their *National Cancer Prevention Policy for Australia *[15]. Structures and programs by which the goals and targets could be achieved were also suggested. The *Health For All Australians *report was intended for the Australian Government, however, it was acknowledged that the proposed changes could only be implemented by collaboration of all stakeholders, including consumers, communities, industry and government. Furthermore, rather than attempting to implement all the strategies in the report, the National Health for All Committee proposed focusing on five national projects. As a result, cancer prevention (primary and secondary), and strategies relating to breast, cervical and skin cancer and tobacco smoking were recommended as initial priorities under the National Program for Better Health. The suggested strategies included:

Breast cancer

• Develop a mammographic screening program

Cervical cancer

• Establish an organised population-based cervical cancer screening program that includes mechanisms and funding to contact the target population at defined intervals, and to ensure the follow-up of women with abnormal smears, quality assurance, program evaluation and epidemiological research.

• Increase participation of eligible women in cervical screening program by call and recall systems, public and professional education, and the provision of alternative services for the taking of smears.

Skin cancer

• Encourage people to stay out of the sun, especially between 10 am and 2 pm

• Use suitable clothing and sunscreen to protect themselves whilst outdoors

• Schedule sporting and other outdoor events before 10 am or after 2 pm

• Lobby to reduce costs of sunscreens, hats, other sun protection products

• Encourage planting of shade trees in public places

• Remove the sales tax on effective sunscreens.

Tobacco smoking

• Improve coordination of smoking control by various government, non-government agencies and public interest groups

• Increase health education about smoking

• Impose restrictions on advertising and promotion of tobacco products

• Impose restrictions on promotion of sports sponsorship by tobacco companies

• Support research into smoking control and causal links with health problems

• Develop coordinated national action to control cigarette advertising

It was estimated that a national cancer prevention project, incorporating research on problems and strategies, planning and development, data collection, monitoring and evaluation, conducted from 1988 to 1992 would cost $12.9 million in total [14]. The five national priority areas proposed were endorsed at the Australian Health Ministers' Conference in 1988 and the National Better Health Program received $39 million over four years to achieve the targets outlined in each of the priority areas [16].

The *Health for All Australians *report represents the first endeavour to assemble a national list of goals and targets for improving health and lessening health inequalities among different population groups in Australia [16]. Several State plans were developed based on the national goals and targets and additional funding was dedicated to these plans [16].

#### Goals & targets for Australia's health in the year 2000 and beyond, 1993

Following from the *Health for All Australians *report, a review was conducted by the Australian Government to help with setting directions, funding health services and improving health in Australia. The subsequent report published in 1993, *Goals & Targets for Australia's Health in the year 2000 and Beyond *[16]: reviewed the progress of the goals and targets published in *Health for All Australians *report, established a framework for setting goals and targets, proposed a new set of goals and targets, and provided options of how the new goals and targets can be achieved in the health system and other sectors.

The new framework recommended a modified range of targets relating to preventable mortality and morbidity, healthy lifestyles, and risks to health. In addition, targets relating to health literacy, health skills, health environments, and the health care system were proposed. In the area of preventable cancer mortality and morbidity, seven specific goals and 16 targets were proposed relating to cancer of the cervix, breast, skin, lung and testis.

Apart from the inclusion of testicular cancer, the cancer goals and targets proposed in this report were largely adapted from those of the *Health for All Australians *report. Whilst the earlier report had a strong focus on risk factors and preventive programs, the *Goals & Targets for Australia's Health in the year 2000 and Beyond *report emphasised the need for an optimal balance of services across the continuum from prevention to palliation [17]. The report also recommended that monitoring and accounting for progress towards achieving targets be the responsibility of a lead agency within the government. The important role of State, Territory and Federal health departments, as well as health professionals, in gaining support from other sectors to effect change within the system was acknowledged.

#### Better Health Outcomes for Australians, 1994

Also in 1993 AHMAC outlined an undertaking to improve both individual and population health by improving health outcomes [17]. In 1994 a joint AHMAC/NHMRC working group was established to select initial health focus areas for national agreement and action. The selection of these initial areas involved extensive consultations with a variety of stakeholders including government and non-government organisations, professional colleges and societies and consumers [17]. The development of goals, targets and strategies was seen as an initial step towards a national approach to improving health outcomes. It was anticipated that the establishment of national health goals and targets would help address inequities in health between different groups in society and provide a framework for the recommendation of strategies that would improve the health outcomes for all Australians [17]. The selection of the health focus areas considered the best available data and evidence on population health, extent of impact on population health, availability and effectiveness of interventions, cost to the community, and potential to reduce health inequalities.

In recognition of the effect of cancer on the Australian population and the potential of reducing morbidity and mortality and improving quality of life by improving cancer control, cancer was one of the initial four health focus areas selected. In the 1994 publication *Better Health Outcomes for Australians *[17], goals, targets and strategies to improve cancer control were outlined and these ranged from prevention to palliation. As an adjunct to the general goals and targets, specific goals and targets were identified for selected 'priority cancers'. Priority cancers were selected on the basis of: burden of illness, preventability, early detection, person years of life lost, increasing incidence, and changing management practices.

Based on these criteria, the seven priority cancers selected were breast cancer, cervical cancer, lung cancer, colon and rectal cancer, melanoma, non-melanoma skin cancer, and prostate cancer.

Government and non-government agencies, health authorities, professional colleges and research centres were charged with implementing the recommended strategies. It was recommended that methods to monitor implementation strategies and report on progress be developed by AHMAC [17].

#### First report on National Health Priority Areas, 1996

In 1995 the Better Health Outcomes Overseeing Committee carried out an evaluation of the national health goals and targets process. Problems relating to the lack of national reporting requirements, too many indicators, and a lack of emphasis on treatment and ongoing management were identified [18]. In 1996, recognising these problems and building on the work of the of the national health goals and targets, the National Health Priority Areas (NHPA) initiative aimed to provide a framework for a national collaborative approach to address five identified priority areas: cardiovascular health, cancer control, injury prevention and control, mental health, and diabetes mellitus.

These NHPA represented conditions with a substantial impact on the community and where a collaborative and focused strategy was thought to be able to achieve substantial health gains [18]. The seven priority cancers identified previously were also focused upon in the NHPA initiative, as considerable health gains were believed to be achievable through prevention and control of these cancers. In the *First Report on National Health Priority Areas 1996 *[18] a selection of 26 indicators in the area of cancer control were outlined. These indicators span the continuum of care and include outcome indicators such as incidence, mortality and five-year survival rates, indicators relating to patient satisfaction, and treatment and process indicators such as screening participation rates, and the creation of hospital based cancer registries. As part of the reporting principles it was agreed that a report on each priority area would be produced, every two years, to provide a summary of the impact of that NHPA on the health of the Australian population [19].

### A historical review – National cancer control strategies and plans from 1996–2005

#### Prevention policies developed by the Australian Cancer Society/The Cancer Council Australia

The Australian Cancer Society (ACS) (now The Cancer Council Australia) conducted a series of expert workshops around Australia and from this produced the 1987 *National Cancer Prevention Policy for Australia *[15]. This outlined what prevention activities should be undertaken, what activities were currently being undertaken, and what research should be conducted to ensure more effective intervention strategies. The report presented a series of goals, targets and strategies in the areas of cancer prevention and early detection and screening for breast, cervical, skin, lung and smoking related cancers.

The report put forward nine national goals, 24 targets and over 30 strategies. In addition, there were several suggestions made relating to quality control of mammography and equity of access. Nine specific research priorities in the areas of cancer causation, primary prevention and screening were identified, along with a recommendation that the Australian Government establish a consultative process to determine a set of priorities for research in cancer prevention.

In 1993 the ACS produced an updated *National Cancer Prevention Policy *[20], setting national goals, targets and strategies related to the prevention of cancer of the skin, breast, cervix, colon and rectum, and smoking related cancers. In total, 13 specific goals and 33 individual targets were proposed. These targets were considered to be achievable by the year 2000.

A further update *National Cancer Prevention Policy 2001–2003 *[21] outlined a strategic approach to reducing Australians' level of risk associated with tobacco, ultraviolet radiation, diet, physical activity, and alcohol. In addition, the prevention policy presents specific strategies and targets for screening for breast, cervical and colorectal cancer.

Although The Cancer Council Australia did not advocate population based screening for prostate cancer or melanoma, the report included strategies for research, evaluation and education in these areas.

The most recent *National Cancer Prevention Policy 2004–2006 *[22] advocates prevention strategies related to tobacco, ultraviolet radiation, diet, physical activity, overweight and obesity, and alcohol. The policy also includes goals and strategies related to screening for breast, cervical and colorectal cancer.

Comprehensive reviews of screening for both melanoma and prostate cancer were conducted and approaches relating to further research, education and development of decision making tools were suggested.

#### Australian government developments: the National Health Priority Areas report: cancer control 1997

In 1997, the first NHPA cancer control report was produced [19]. It identified major issues relating to the priority cancers and opportunities to improve cancer control in Australia, relating to the seven priority cancers and the continuum of care. The report also identifies other opportunities for improvements in cancer control including:

• The role of general practitioners (GPs) in prevention and early detection

• Best practice guidelines

• Multidisciplinary care

• Palliative care

• Psychosocial care

• Supportive care

• The development of consumer networks

• Special populations

• Familial cancers

• Research, and

• Data collection

In this first NHPA report on cancer control the concept of using a framework for fostering a national approach to cancer control is outlined. The framework encompasses different cancer types, stages along the continuum of care and other health system activities that may be relevant to cancer control. It was proposed that using such a framework would facilitate the identification of gaps and problems which could be dealt with, leading to further improvements in health outcomes [19]. This 1997 NHPA report was submitted to the Federal and State Health Ministers, who had the authority and responsibility for implementing and progressing the opportunities for improvements in cancer control in Australia.

#### Combined government and non-government approaches: cancer control towards 2002

The National Cancer Control Initiative (NCCI) was set up in 1997 as an expert advisory group to the Australian Government and other key groups. It was funded by the Australian government but operated and supported by The Cancer Council Australia and The Cancer Council Victoria. As its first task, the NCCI undertook a process in 1997 to identify a set of consensus-based priorities for cancer control [23]. The priority cancer control areas outlined in the 1996 NHPA report [18] and the 1997 NHPA Cancer Control report [19] were considered and 36 topic areas were identified, and an expert working party was set up to consider each of these and propose actions. This process produced a list of 276 proposed actions. These were then assessed in terms of their ability to achieve change within a five year time period, and consideration was also given to health impact indices, potential of the actions to reduce the burden of illness, levels of evidence, cost-effectiveness, and potential to reduce inequity. Workshops were conducted in each State and Territory, and further questionnaires sent to stakeholders. By these processes, the number of proposed actions was refined to 21 recommended actions, 13 of which were recommended as priority actions. These consensus-based proposed actions were published in *Cancer control towards 2002 *[23] and are listed in Table 1. The 13 actions for priority implementation spanned the areas of primary prevention, population-based screening and early detection, and treatment. For each action recommended for priority implementation, the report outlined the costs, roles and responsibilities and timelines required to implement the actions. These proposed actions were discussed with the Commonwealth Minister of Health and Ageing, and with some modifications, were the basis of the work plan for NCCI over the next few years.

**Table 1:** Priorities for cancer control developments likely to have benefits within five years, identified by consultations by the National Cancer Control Initiative (1997)

Primary prevention		
1*	Tobacco	Preventing tobacco-related cancers: strengthen tobacco control measures
2	Skin cancer	Reducing risk

Population-based screening and early detection		

3	Breast cancer	Improving BreastScreen Australia
4	Breast cancer	Promoting prompt diagnosis
5	Cervical cancer	Improving Pap smear programs
6	Cervical cancer	Handling Pap smear results
7*	Colorectal cancer	Developing faecal occult blood testing: develop demonstration program and evaluation, and produce evidence-based guidelines
8*	Prostate cancer	Rationalising prostate-specific antigen testing: discourage inappropriate use of PSA tests and develop educational program for GPs
9*	Skin cancer	Improving diagnostic skills: design and evaluate programs to improve GP's skills in diagnosis of early skin cancer

Treatment		

10*	Guidelines	A national approach: identify priority areas and develop and disseminate evidence-based clinical guidelines
11*	Multidisciplinary care	Evaluation and facilitation: identify benefits and costs of multidisciplinary care and improve consultations especially in rural and remote areas
12*	Palliative care	Filling gaps: develop national strategies and research on co-ordinated palliative care
13*	Prostate cancer	Dealing with treatment uncertainties: assess the treatment and outcome of early and advanced prostate cancer
14*	Psychosocial care	Defining, implementing and monitoring: define appropriate psychosocial care, develop a strategy, and establish national consumers' forum in cancer control

General		

15*	General practice	Promoting participation in cancer control: improve preventive, screening and early detection, and cancer management, in general practice
16	Equity	Implementing culturally relevant cancer control measures
17	Consumer	Facilitating involvement
18*	Research	Continuing the national commitment: maintain and extend
19*	Familial cancers	Organising education and resources: develop educational approaches, link genetic registries, and promote research
20*	Data collection	Meeting urgent national needs: develop a national standard for clinical data registries, add staging information to registries
21	Clinical trials	Encouraging participation of doctors and patients

*The 13 out of 21 proposed actions recommended for priority implementation. Table reproduced from *Cancer Control Towards 2002 *[23]

#### Priorities for Action in Cancer Control 2001–2003

The National Cancer Strategies Group (CSG), a sub-committee of the National Health Priority Action Council, was established in mid 1998 to develop a National Cancer Control Plan based on the NCCI's priority setting process. The CSG is a committee of the Australian Government Department of Health and Ageing, but with a non-government chair, representatives of consumers, professionals, and non-government bodies, of the NCCI and NBCC, and representatives from the States and Territories. A workshop was held in 1999 to review and update the priorities identified in the previous NCCI report. The NCCI priorities were accepted as still valid, with some additions and modifications. In a new development, several incremental proposals (e.g. implementation of colorectal screening) and two decremental proposals (e.g. performing cervical screening less frequently) were selected for assessment by Program Budgeting and Marginal Analysis (PBMA) [24]. This process combines assessment of scientific evidence with a marginal cost-benefit analysis and explicit consideration of other factors such as equity and acceptability. Thirteen priority actions for implementation were identified and published in *Priorities for action in cancer control 2001–2003 *(PACC) [6]. The priority actions were in the areas of: prevention, screening and early detection, treatment, and supportive and palliative care.

Seven of the 13 priority actions were included in the PBMA analysis and all seven actions were assessed as cost effective, with three actions having net cost savings (programmes of smoking prevention, sun protection, and increasing fruit and vegetable intake). Two recommendations were for cost saving reductions in activity (doing cervical cancer screening less frequently and from a later age). This report included 'second stage filters', giving explicit consideration to equity implications, levels of scientific evidence, the size of the health problem, and the acceptability and feasibility of the changes recommended. The priority actions addressed seven of the eight NHPA priority cancers (non-Hodgkin lymphoma was added in 1998 as the eighth priority cancer [25]).

The priority actions complement other national cancer control actions, namely those identified in the NHPA initiative [19] and the NCCI plan [23] and a review was proposed on a three to five yearly basis. The PACC report did not include an implementation plan for the priority actions, but recommended that an implementation plan be developed in consultation with key stakeholders.

#### Optimising Cancer Care in Australia

The PACC report provided an ambitious list of new national cancer control priority actions, supported by a strong evidence base and sound economic analysis. However, there were constraints on the scope of the PACC report. In particular, it did not address issues of routine care. In 2002, The Cancer Council Australia, the Clinical Oncological Society of Australia (COSA) and the NCCI jointly developed a consultative report, which aimed to identify systematic problems, barriers and failings of the current system of cancer care in Australia, focus on key priorities and be based on consultations with key organisations and individuals in the field of cancer care, and address pertinent issues to provide a framework and recommendations for policy development. The report *Optimising Cancer Care in Australia *(OCCA) [3] was developed in three main stages. The first stage, undertaken by an independent consultant, involved reviewing information and existing research on cancer care in Australia and internationally and conducting interviews with a wide variety of stakeholders around Australia to identify key themes, issues and opportunities for optimising cancer care in Australia. In the second stage, a stakeholder workshop was convened by the NCCI and the key themes were explored and refined into a workable number of key issues. A draft report was prepared, and stage three of the development process involved circulating the draft document for public comment. A second workshop was held and the report was revised following input from all interested stakeholders. The final report was launched in February 2003. Several major consistent findings emerged from the consultations. These included:

• That while survival rates from cancer in Australia are very good by world standards, there was the potential to produce much better outcomes through organisational reform of the way in which cancer services are delivered, and

• As a nation we are under-investing in cancer relative to the magnitude of the problem and many reforms could be achieved without a large increase in the health care budget.

The report's opening statement was *"There is a strong conviction held by consumers and cancer care providers that Australian cancer services can be, and must be, improved substantially. Survival, quality of life, and the 'cancer journey' would greatly improve if everyone received optimum treatment."*

There was strong consumer input into the report. Many consumers felt that the cancer care system did not provide what they felt was necessary and reasonable. Priorities for consumers included: patient focused, coordinated multidisciplinary care, an end to the cancer care 'referral lottery', reasonable access to evidence-based quality care including clinical trials, and support throughout the cancer journey.

Based on these consultations and research, 12 key recommendations and 19 action items were proposed. These related to four key areas of change: models of cancer care, quality of cancer care, resource issues in cancer care and improving the delivery of cancer care. The recommendations emphasised integrated multidisciplinary care, and care throughout the cancer journey, including palliative and supportive care and improved consumer access to information. There were recommendations for the development of an accreditation system for cancer care services, improved access to clinical trials and to psycho-oncology services and to new and accepted pharmaceuticals, implementation of already existing workforce plans for the oncology workforce and for radiation oncology, revision of the system of support for the travel of patients and carers to receive care, and special attention to equity of access, especially in Aboriginal peoples. The recommended strategy for implementation was to form a 'National Taskforce' on cancer to oversee and drive the reform process, supported by adequate funding.

The recommendations were intended for consideration by the Commonwealth Minister for Health and Ageing in concert with State and Territory health authorities.

The OCCA report has helped in stimulating major action in cancer control in Australia at national and State levels, and is one of the key documents on which the *National Service Improvement Framework for Cancer *[26] is based.

#### National Service Improvement Framework for Cancer

In October 2002, AHMAC agreed to develop National Service Improvement Frameworks (NSIF) for the seven national health priority chronic conditions. The framework development process was to be overseen by the National Health Priority Action Council and represent a joint initiative between State and Territory Governments. The Frameworks aim to:

• Prevent and limit the progression of these chronic conditions

• Slow the onset of the complications that can cause severe disabilities

• Reduce preventable hospital admissions

• Reduce variations in care that are provided

- By different clinicians and health services

- To people from metropolitan, regional, rural and remote areas; and

- To disadvantaged groups

**Figure 1:**
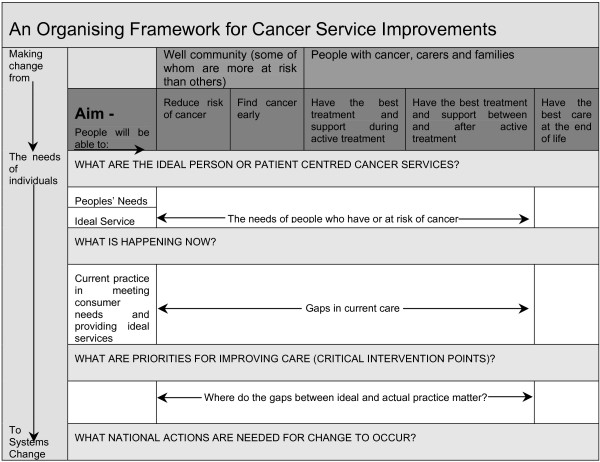
National Service Improvement Framework for Cancer. Reproduced from National Service Improvement Framework [26] with permission from the Australian Government Department of Health and Ageing.

The NSIFs are designed to be high level guides for health services and are intended to inform consumers, clinicians, planners and designers, policy makers, funders and providers and professionals and managers [26]. Specifically the frameworks are designed to foster adoption of processes, but they are not intended to replace clinical practice guidelines, accreditation, clinical audit or benchmarking [26]. Moreover, the NSIF are meant to complement State and Territory plans and frameworks. The NSIF for cancer *"outlines what all Australians with, or at risk of, cancer should expect to be provided through the Australian health care system, irrespective of where they live" *[26]. The framework for cancer is shown in Figure 1. The framework extends over five main phases, which span the continuum of care from reducing risk to care at the end of life, and addresses issues ranging from the needs of individuals to systems change. However, the framework does not include specific strategies relating to the NHPA cancers or special populations. Nineteen critical intervention points fit into this framework and have been chosen through a process of identifying the information needs of the well community about a chronic disease, the needs of people with a chronic disease, optimal system or service response, gaps between optimal system or service and current system and service delivery, and gaps which represent the best opportunities for gains in health outcomes and improvement at the system level [26]. The 19 critical intervention points span the continuum of care and include interventions in areas of: reducing risk, finding cancer early, management and support during active treatment, management and support after and between periods of active treatment, and care at the end of life if cancer is not curable.

In addition, there are eight priority actions that underpin the critical intervention points. The priority actions include establishing:

• Integrated and networked cancer services

• Accreditation for cancer services and credentialing of practitioners

• Funding structures to support multidisciplinary care

• Approaches to monitor cancer control

• Provision of consumer information about cancer risks, prevention, early detection, diagnosis and treatment and supportive care

• Support for primary care providers to provide appropriate assessment of risk

• Implementation and evaluation of culturally appropriate programs to improve cancer control, and

• Review gaps in research and opportunities at least every three years

It has been proposed that if these priority actions were implemented by the Australian and State or Territory governments, the foundation for many of the changes identified in the framework would be in place [26]. The NSIF for cancer report does not include an implementation plan. Rather, the implementation plan will be developed later with input form State and Territory governments, non-government organisations, professional colleges, consumer organisations, and other key stakeholders [26].

#### Strengthening Cancer Care

Prior to the federal election in 2004, the Coalition Government released a pre-election document *Strengthening Cancer Care *[27]. This document outlined a series of initiatives to improve cancer care in Australia including supporting Australians living with cancer and the professionals who care for them, enhancing screening and prevention efforts in bowel and skin cancer, smoking in pregnancy, better access to Pap smears for cervical cancer, ensuring better coordination of the national cancer effort, and more research funding dedicated to cancer and cancer care.

In the 2004–05 federal budget it was announced that $189.4 million would be made available over a five-year period to 2008–09 for the *Strengthening Cancer Care *initiative. The funding will support the further development of existing services and programs and the establishment of new initiatives. In particular, this funding will be allocated to:

• Developing and implementing training courses for cancer nurses

• Improving professional development for cancer professionals, counsellors and general practitioners

• Developing and implementing mentoring for regional cancer services

• Improving support for those newly diagnosed with breast cancer

• Increasing cancer research

• Enhancing cancer screening and awareness

• Supporting cancer clinical trials

• Building cancer support groups

• Providing medical benefits schedule (MBS) eligibility for a magnetic resonance imaging (MRI) unit at Sydney's Children's Hospital

• Supporting children with cancer and their families

• Enhancing palliative care programs

• Improving the early detection and management of breast cancer

• Establishing a new national cancer agency, Cancer Australia

• Establishing a national research centre for asbestos related diseases

• Providing additional radiation therapy internships and undergraduate places

• Redeveloping the children's cancer ward at Royal Children's Hospital in Melbourne, and

• Evaluation of the *Strengthening Cancer Care *initiative at the end of the five-year funding cycle.

### State and territory cancer control strategies and plans

Many of the States and Territories in Australia are developing their own local cancer control plans. At the time of this review (December 2005), all States and Territories, with the exception of the Northern Territory, had produced or were developing State based plans. By December 2005, only Victorian and New South Wales had published their cancer control plans.

#### Victoria

In 2003, *A Cancer Services Framework for Victoria *[28] was produced. The proposed Victorian framework is built around evidence-based specific standards of care for the 10 most frequently occurring cancers. The framework also includes the creation of integrated cancer services (ICS) both in metropolitan Melbourne and rural and regional centres. It is envisaged that the ICS would consist of groups of hospitals and health services that would be responsible for the provision of cancer services to people in that geographical area. The report proposes 47 recommendations relating to service delivery, information, benchmarks and quality improvement, proposed Victorian cancer services framework, and future directions for the Peter MacCallum Cancer Institute.

The *Cancer Services Framework for Victoria *together with the Victorian Government's *Fighting Cancer Policy *[29] forms the basis for the restructuring, integration, and development of cancer services and research in Victoria [13]. To assist in accomplishing these reforms the Victorian government has formed a Ministerial Taskforce for Cancer. The Ministerial Taskforce has recently produced *Towards better care for all cancer patients *[13] a cancer action plan for 2005–2006. In this report the Taskforce have identified three priority areas, clinical services, research, and data/information. A working group is charged with providing leadership and achieving set goals in each priority area. Outlined within each area are several priority areas of focus and key deliverables for 2005–2006. In the area of clinical services areas of focus, include:

• Developing an integrated approach to cancer care

• Developing care through ten tumour streams, and

• Developing statewide approaches to service improvement.

The priority areas for the research working group are:

• Developing a strategic approach to cancer research

• Developing and supporting cancer research, and

• Translating knowledge from research into care and services.

Finally the three main areas for the data/information working group are:

• Improving data collection and information management

• Developing better reporting structures, and

• Establishing benchmarks to lead best practice.

#### New South Wales

In New South Wales the Cancer Institute NSW produced the *NSW Cancer Plan 2004–2006 *[12] in mid 2004. This comprehensive plan builds on previous State initiatives including the *Clinical Service Framework for Optimising Cancer Care in NSW *[30] and was developed with wide stakeholder input. The plan aims to:

• Define the strategic principles for the future development and acceleration of effective cancer control in NSW

• Develop goals for cancer control that will substantially improve outcomes, and

• To develop high-priority programs that will achieve these goals and thereby accelerate improvements in cancer survival, reduce cancer incidence, better support patients and their carers and better inform the community and other important groups.

In line with these objectives the *NSW Cancer Plan *outlines 33 specific goals in 10 strategic areas. These areas are:

• Coordination of cancer control

• Cancer prevention and early detection

• Cancer service provision-the patient's journey

• Special issues in cancer care

• Cancer information

• Cancer education

• Cancer workforce

• Cancer research

• Cancer fundraising, and

• Quality, evaluation and accreditation.

In addition, there are 33 key results and 79 outcomes relating to the 33 goals identified in the report. The implementation of the plan will be developed following further consultation.

## Discussion

From the mid 1980's onwards, several major national and State based reports have addressed issues of cancer control and cancer care in Australia. These have been produced in response to the recognised impact and importance of cancer in Australia, and the potential gains that can be made in morbidity and mortality through effective cancer control and addressing inequities in cancer care. These reports identify priority cancers and strategies judged to have the potential to improve cancer health outcomes. The importance of prevention and strategies to reduce risk are the focus of several reports. For example, goals, targets and strategies relating to tobacco smoking and to skin cancer were outlined in the *Health for all Australians *report [14] and specific prevention strategies relating to tobacco and ultraviolet radiation exposure are still prominent in the latest *National Cancer Prevention Policy *[22]. The reports have reflected current evidence and research; for example, the recommendations supporting the implementation of population-based bowel cancer screening using faecal occult blood testing [22].

There are now eight NHPA priority cancers. The PACC report [6] and the NHPA Cancer Control report [23] identify priority actions and cancer control strategies that relate to the original seven priority cancers, lung, colorectal, melanoma, non-melanoma, breast, cervical, and prostate cancer. Although non-Hodgkin lymphoma has been added as a priority cancer on account of its considerable and increasing incidence, no specific strategies have been identified for it. Several reports including OCCA and NSIF take an inclusive approach applying to all cancer, with goals and targets relating to the continuum of care [16-19,23,6,26] and emphasising multidisciplinary and integrated care. In some instances the reports also outline structures, process and frameworks by which the goals could be achieved [14] and recommend that a national collaborative approach to cancer control be further developed [18,19,26,28].

The development of cancer control plans and strategies in Australia has encompassed increasing involvement of stakeholders. The *Better Health Outcomes for Australians *[17], OCCA [3], and PACC [6] reports are examples where wide stakeholder input and consultations were used for the identification of initial priority areas and the development of recommendations. Stakeholder input into the development of these plans and strategies is important. Not only does stakeholder input provide an opportunity to identify a wide range of issues from different sectors but it also has the potential to foster ownership of recommendations and can thus facilitate intersectoral participation and collaboration in implementation of cancer control plans.

Weaknesses in the reports relate to the lack of implementation plans, the absence of dedicated funding for implementation, too few economic analyses supporting priority actions and strategies, and little or no evaluation of uptake or impact of cancer control plans. Perhaps the central weakness is that those producing the plans are not the same as those who have the authority to carry them out: for example, most of the early plans came from the federal government and the non-government sectors, but most specialist cancer care is funded and controlled at state level. Even the recently formed NSW Cancer Institute has control over only a very small part of NSW health expenditure on cancer. So, all these plans depend for their implementation on other groups who may have other priorities. This is in contrast with, for example, the British NHS, which both produces strategic plans and implements them.

The number of cancer specific recommendations, actions or goals presented in the reports ranges from 13 to 46. Presenting too many recommendations or actions has the potential to weaken focus and reduce the impact on cancer outcomes. A well-structured implementation plan may overcome these problems: however, only a few reports contained a comprehensive plan. Several reports acknowledged the need for an implementation plan or identified people or groups who would be responsible for the implementation and monitoring of strategies [16,17,19,6,3,26]. However, implementation strategies were often not formally developed for these reports. There has generally not been a clear assignment of responsibility and accountability for developing, implementing and monitoring cancer control strategies, particularly on a national basis. Funding of implementation is also crucial for progressing cancer control strategies and changing health outcomes. Several reports have acknowledged the importance of collaboration between States and Territories and the need for collaboration between different organisations, professional bodies and stakeholders to implement strategies and foster change [14,16,23,26]. However, governments cannot rely on the benevolence of other stakeholders to implement strategies and achieve national goals and targets without financial support.

Some reports clearly indicate the financial costs associated with undertaking priority actions and implementation strategies [14,23,6]. The NCCI's *Cancer control towards 2002 *[23] report outlines costs required to implement actions and the PACC report [6] undertook a PBMA to identify the cost effectiveness of seven of the 13 priority actions. However, many of the other proposed priorities in the cancer plans have not considered economic costs. While the strategies proposed in the reports have the potential to impact on cancer in the Australia community, whether the associated impact is at an acceptable financial cost is often unclear.

The recommendations, strategies and goals proposed in the reports relate to the continuum of care, specific cancers, the development of supportive framework and process issues. While the progress of individual strategies and the attainment of particular goals can be identified and assessed, to date, none of the reports have ever been evaluated for the extent of implementation of proposed actions or their overall impact on health outcomes at a national level.

The objective assessment of the outcomes of health strategies becomes more difficult as it moves from very specific strategies such as a change in treatment regimen, to the more general strategies and changes in health services structures. The specific components of many of these strategic approaches have been or can be evaluated; for example, the impact of screening programs on disease and the effects of the introduction of multidisciplinary care in specific situations are amenable to assessment. Whether overall national strategic programs can be assessed is more questionable, if only because they are not introduced in a controlled fashion. Overall outcomes such as cancer survival rates and mortality, participation in screening programs, measures of consumer satisfaction, and studies to show the comparability of actual clinical practice with best practice where that has been defined, can all contribute to the assessment.

## Conclusion

The goal of cancer control is to reduce the impact of cancer on the community and improve health outcomes. National and State cancer plans have identified important opportunities for doing this. Indeed, over time, certain recommendations, goal and targets have been successfully implemented leading to improved health outcomes. Developments in new technologies, diagnosis and treatment, and the identification of risk factors constantly shift the goal posts and cancer control plans need to be flexible and updated on a regular basis to revise and re-prioritise goals. The limited evaluation of cancer control plans reduces our ability to assess the overall success of these plans. Overall, these national and state plans have outlined ambitious agendas for change. Through appropriate structures, leadership, and financial support for implementation and evaluation, these reforms have the potential to significantly reduce the impact of cancer in the Australian community.

## Abbreviations

AHMAC Australian Health Ministers' Advisory Council

NHMRC National Health and Medical Research Council

NHPA National Health Priority Areas

ACS Australian Cancer Society

NCCI National Cancer Control Initiative

CSG Cancer Strategies Group

PBMA Program Budgeting and Marginal Analysis

PACC Priorities for Action in Cancer Control 2001–2003

TCCA The Cancer Council Australia

COSA Clinical Oncological Society of Australia

OCCA Optimising Cancer Care in Australia

NSIF National Service Improvement Frameworks

ICS Integrated Cancer Services

## Competing interests

Mark Elwood, Cleola Anderiesz, David Hill

Mark Elwood was a member of the National Cancer Strategies Group.

Cleola Anderiesz has declared no competing interests

David Hill is a member of the Ministerial Taskforce for Cancer in the State of Victoria.

## Authors' contributions

CA drafted the manuscript. ME and DH had input into the content and revision of the manuscript. All authors read and approved the final manuscript.
